# Comparative evaluation of *Aspergillus niger* strains for endogenous pectin-depolymerization capacity and suitability for d-galacturonic acid production

**DOI:** 10.1007/s00449-020-02347-z

**Published:** 2020-04-23

**Authors:** Dominik Schäfer, Kevin Schmitz, Dirk Weuster-Botz, J. Philipp Benz

**Affiliations:** 1 Department of Mechanical Engineering, Institute of Biochemical Engineering, Technical University of Munich, Boltzmannstr. 15, 85748 Garching, Germany; 2 Holzforschung München, Wood Bioprocesses, TUM School of Life Sciences Weihenstephan, Technical University of Munich, Hans-Carl-von-Carlowitz-Platz 2, 85354 Freising, Germany

**Keywords:** *Aspergillus niger*, Agricultural residues, Sugar beet pulp, Pectinase, d-galacturonic acid

## Abstract

Pectinaceous agricultural residues rich in d-galacturonic acid (d-GalA), such as sugar beet pulp, are considered as promising feedstocks for waste-to-value conversions. *Aspergillus niger* is known for its strong pectinolytic activity. However, while specialized strains for production of citric acid or proteins are well characterized, this is not the case for the production of pectinases. We, therefore, systematically compared the pectinolytic capabilities of six *A. niger* strains (ATCC 1015, ATCC 11414, NRRL 3122, CBS 513.88, NRRL 3, and N402) using controlled batch cultivations in stirred-tank bioreactors. *A. niger* ATCC 11414 showed the highest polygalacturonase activity, specific protein secretion, and a suitable morphology. Furthermore, d-GalA release from sugar beet pulp was 75% higher compared to the standard lab strain *A. niger* N402. Our study, therefore, presents a robust initial strain selection to guide future process improvement of d-GalA production from agricultural residues and identifies a high-performance base strain for further genetic optimizations.

**Electronic supplementary material:**

The online version of this article (10.1007/s00449-020-02347-z) contains supplementary material, which is available to authorized users.

## Introduction

Global academic and industrial efforts to improve the sustainability of industrial processes for the modern bio-economy have sparked interest in the utilization of feedstocks that are economically viable, non-food grade, and do not compete with food resources [6, 23, 26, 44, 49, 51, 58]. As a result, agricultural waste streams have gained momentum in the recent years (reviewed by Amoah et al*.* [3]). Notably, downstream fermentation products derived from pectin-rich biomass are met with industrial interest by the plastics, cosmetics, and food industries, as recently reviewed by Kuivanen et al*.* [28], Schmitz et al*.* [46], and Richard and Hilditch [43].

Besides harsh thermo-chemical treatment and hydrolysis approaches [12], fermentation broths of natural pectin-degrading microorganisms containing complex enzyme mixtures can be used to liberate the constituent saccharides, such as d-galacturonic acid (d-GalA), which is the main backbone sugar of pectin. The filamentous saprophytic fungus *Aspergillus niger* is a well-known microorganism for pectin utilization and depolymerization as well as a well-established industrial workhorse with multiple applications in the production of enzymes, citric acid, and other organic acids [9, 31]. Pectinases from *A. niger* contribute to a global multi-billion dollar market for biomass-degrading enzymes [9, 31, 47] with applications ranging from fruit, vegetable, and juice processing to textile and paper treatment [22] as well as saccharification for bioethanol production [11, 50, 56]. However, with today’s perception of d-GalA shifting from an inevitable component of complex biomass feedstocks to a target product for subsequent fermentations, more versatile strains that can achieve higher d-GalA yields are needed to extend the range of commercially available saccharification enzymes, as outlined in a recent white paper on the current challenges of research on filamentous fungi in the context of a sustainable bio-economy [31].

**Fig. 1:**
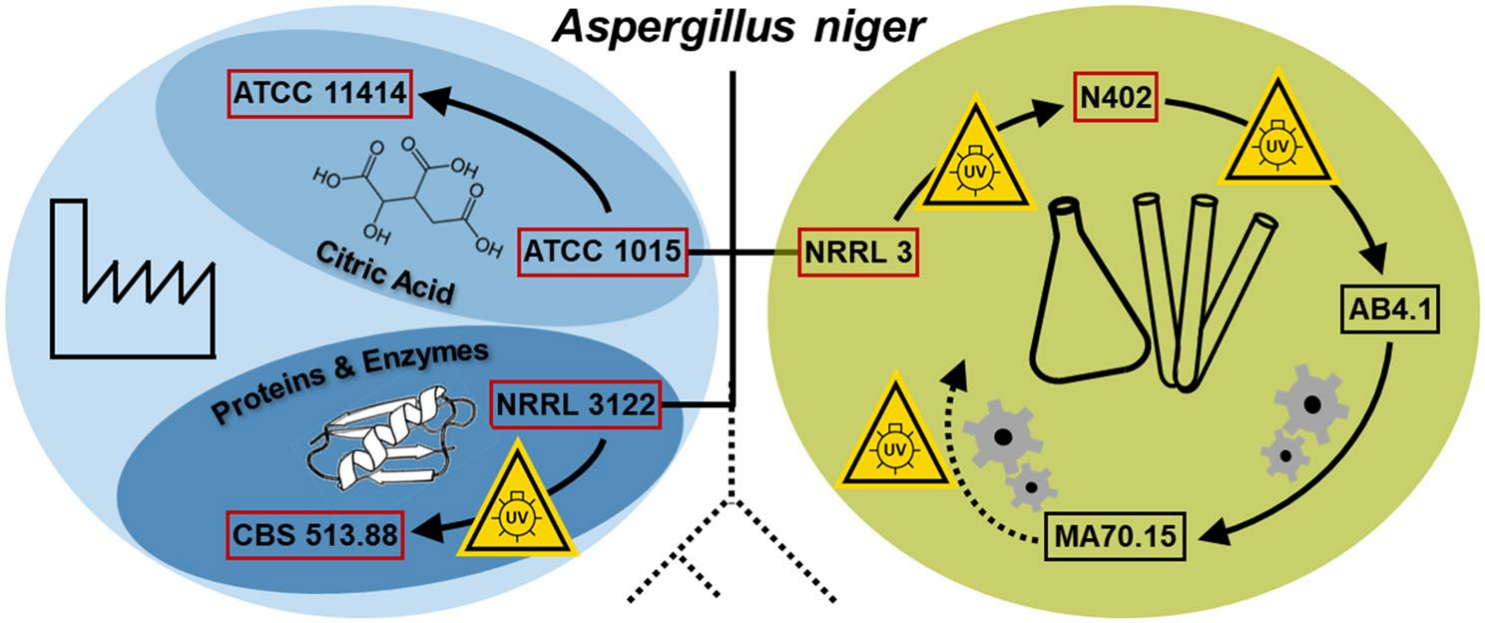
Highly relevant lineages of *A. niger* and their primary applications. Schematic overview listing selected but representative members of openly available and related *A. niger* strains based on phylogeny [55]. Depicted are commonly used lab strains (green, right) vs. the industrially adapted strains (blue, left) including the citric acid producer clade (light blue) and the more distantly related enzyme producers (dark blue). Yellow signs indicate instances of UV mutagenesis, grey gear wheels symbolize targeted genomic engineering steps, and red boxes indicate the strains used in this study (color figure online)

To enable efficient industrial-scale applications, ideal base strains need to be identified for specific tasks. For *A. niger*, several lineages have been identified and adapted for specific purposes. The most cited lineages of *A. niger* encompass three main clades, namely: (i) strains adapted for easy handling and genetic manipulation in the laboratory environment, which are based on *A. niger* NRRL 3 (CBS 120.49, ATCC 9029); (ii) strains for improved citric acid production, based on *A. niger* ATCC 1015 (NRRL 328, CBS 113.46); and (iii) strains for protein production and secretion, based on *A. niger* NRRL 3122 (CBS 115989; ATCC 22343) (Fig. 1). To the best of our knowledge, no thorough comparison of pectinase activity between these available and highly cited *A. niger* strains has been conducted so far. While the commercial availability of *A. niger* pectinase cocktails indicates that the industry has established strains for pectinase production, almost no information on their specific origin is publically accessible. In the academic field, numerous studies on optimization of fermentation conditions for pectinase production with various strains have been published (e.g., [1, 18, 42]). However, these do not allow for direct performance comparison of individual strains due to varying study designs and fermentation conditions. Furthermore, thorough comparisons of different strains under reproducible conditions are scarce in the literature and compromised by limited morphology control (due to execution in shake flasks, for example, instead of controlled stirred-tank bioreactors) or poor description of strain origins (e.g., [17, 25]).

As outlined in Fig. 1, ancestral strains have been specifically isolated due to their improved citric acid production characteristics (*A. niger* ATCC 1015) or extracellular glucoamylase activity (*A. niger* NRRL 3122), respectively, and further optimized via sub-culture isolation [39] or UV mutagenesis [4, 53]. Similarly, multiple successive rounds of UV mutagenesis on the original wild-type (WT) isolate *A. niger* NRRL 3 [7] have resulted in a short conidiophore phenotype in strain *A. niger* N402 [8] and the derived strain most commonly used for laboratory-based genetic analysis, *A. niger* AB4.1, containing the *pyrG* auxotrophic marker [19, 52]. However, those mutagenic treatments likely gave rise to additional background mutations influencing gene regulation and impacting diverse phenotypic traits. Additional targeted engineering steps for improved homologous recombination (*A. niger* MA70.15) [32] or the introduction of auxotrophic markers [16, 35] have further expanded the genetic toolbox in the laboratory. At the same time, however, these may induce stress or alter intracellular regulation and, hence, divert these strains further from their wild-type physiological constitution [41], which might impair fungal productivity and, hence, should be considered in strain selection.

From a genomics perspective, genomic sequences are openly available for *A. niger* strains CBS 513.88 [38], ATCC 1015 [4], and NRRL 3 [2]—the latter with a recent advance in annotation [45]—as well as for three additional isolates [55]. Comparison of intra-species genomic data revealed the cause of some of the observed phenotypic differences between the strains, such as overproduction of glucoamylase in *A. niger* CBS 513.88, which was due to an additional glucoamylase gene acquired by horizontal gene transfer [4, 55]. Transcriptional profiling, however, also indicated that the regulatory networks between different strains are already highly divergent and cannot be explained purely by genomic observations [4]. Additionally, fungal productivity for different purposes depends on morphology control in submerged cultures [10, 24, 54, 57].

Accordingly, none of the current data sets provide enough information to predict superiority of any available strain for the production of pectinases.

The importance of pectin and pectin-derived sugars, as well as the predominance of *A. niger* in pectinase production processes, however, warrant a systematic comparison of strains under controlled and highly reproducible conditions to identify efficient host strains for larger scale pectinase production. Therefore, a total of six strains (red boxes in Fig. 1) were selected for comparison of endogenous pectinase activity based on five key prerequisites: (i) relevance to the field based on the number of publications using these strains, (ii) availability of the genomic sequence (or that of a very closely related strain) as a premise for successive genetic optimization, (iii) absence of auxotrophic markers, to avoid phenotypic differences due to mutations in central metabolism, (iv) absence of any targeted engineering of elements regulating pectinase expression, to avoid distortion of the underlying endogenous pectinase capacity, and (v) classification as biosafety level 1 to allow for universal handling.

By obtaining data on sporulation efficiency, total protein secretion, total and endo-specific polygalacturonase (PGase) activity, as well as morphology in submerged culture, this study provides essential insights into selection of suitable base strains for pectinase production. Working with controlled stirred-tank bioreactor fermentations after the initial pre-selection, we applied a robust and reproducible methodology rarely employed in phenotypic comparisons of fungal strains, resulting in the identification of a superior *A. niger* strain and potential chassis for additional genetic optimization to boost d-GalA release from complex pectinaceous residues.

## Materials and methods

### Strains, inoculum preparation, and cultivation medium

*A. niger* strains ATCC 1015, ATCC 11414, and NRRL 3122 were obtained from the NRRL collection, and NRRL 3, N402, and CBS 513.88 were obtained from the group of Arthur Ram at Leiden University. Fungal spores were grown on 39 g L^−1^ potato extract glucose agar (Carl Roth GmbH + Co. KG, Karlsruhe, Germany) supplemented with 10 g L^−1^ yeast extract and 1 × trace elements solution, referred to as rich complete medium. After 120 h at 30 °C, spores were harvested using sterile 0.89% NaCl solution with 0.05% Tween 80.

All experiments involving submerged fungal cultivation were carried out in 2% (w/v) pectin minimal medium containing (L^−1^): 20.0 g pectin C, 6.0 g NaNO_3_, 1.5 g KH_2_PO_4_, 0.5 g KCl, 0.5 g MgSO_4_ · 7 H_2_O, 1 mL trace-element solution, and 1 mL PPG P2000 (antifoam, only if cultivated in stirred tank fermenter). The trace-element solution was prepared as (L^−1^) 10 g EDTA, 4.4 g ZnSO_4_·7 H_2_O, 1.01 g MnCl_2_·4 H_2_O, 0.32 g CoCl_2_·6 H_2_O, 0.315 g CuSO_4_·5 H_2_O, 0.22 g (NH_4_)_6_Mo_7_O_24_·4 H_2_O, 1.47 g CaCl_2_·2 H_2_O, and 1 g FeSO_4_·7 H_2_O [5].

### Cultivation conditions

#### Shake flask cultivation

The fungi were grown in 250 mL flasks without baffles containing 25 mL of the 20 g L^−1^ pectin minimal medium at 250 min^−1^ (25 mm shaking throw) and 30 °C for 96 h. The initial pH was set to 4.5 and the medium was inoculated to a spore density of 10^9^ spores L^−1^. Strains were grown in triplicates. Data were statistically evaluated by applying an analysis of variance (one-way ANOVA) followed by a Tukey’s post hoc test using the software Origin (OriginLab). Differences among the mean activity measurements were calculated at a significance level of 0.05.

#### Bioreactor cultivation

A 7 L stirred-tank bioreactor equipped with three baffles and three six-blade Rushton turbines (Labfors, Infors-HT, Bottmingen, Switzerland) was used during all cultivations. All processes were performed equally under the following conditions. Three liters of the 20 g L^−1^ pectin mineral medium was inoculated to 10^9^ spores L^−1^. Temperature was kept constant at 30 °C. The pH was controlled to a set-point of pH 4.5 by the addition of either 1 M H_2_SO_4_ or 3 M KOH. Batch processes were carried out for 86–90 h. To prevent the initial spore loss, the stirred-tank bioreactor was not aerated and only slowly mixed at 250 min^−1^ (~ 0.130 W L^−1^ [21]) during the first 6 h of batch cultivations [34]. Afterwards, the stirrer speed was set to 700 min^−1^ (~ 1.625 W L^−1^ [21]) and aeration to 0.2 vvm, which was also sufficient to keep the dissolved oxygen concentration above 30% air saturation during all cultivations conducted. Additionally, exit-gas composition (O_2_, CO_2_) was monitored (EasyLine, ABB, Zürich, Switzerland).

### Biomass dry weight concentration

Biomass dry weight was determined by filtering a known volume thought pre-dried and pre-weighed filter paper (Whatman No. 1 and 5). The collected biomass was dried at 90 °C to constant weight and reweighed. The determination of the biomass dry weight was performed in triplicate and expressed as the mean with standard deviation of the measurements.

### Morphological characterization

Microscopic images for morphological characterization were taken with an Axioplan microscope (Carl Zeiss AG, Jena, Germany) at 1.25 × magnification directly after sampling after 9, 12, 19, 36, and 88 h of the batch cultivation. The microscope was equipped with a 3.3-megapixel Axiocam ICc3 microscopy camera (Carl Zeiss AG, Jena, Germany).

### Protein concentration of culture supernatant

Protein concentration of the culture supernatant was determined using the Coomassie (Bradford) Protein Assay Kit (Thermo Scientific) according to the manufacturer's specifications. Each sample was diluted with 0.1 M sodium citrate buffer pH 4.5, mixed with the Bradford reagent, and incubated for at least 10 min at room temperature. Afterwards, the absorbance at 595 nm was measured with a multimode microplate reader (Infinite M200, Tecan, Männedorf, Germany). Bovine serum albumin was used as the standard. The determination of the protein concentrations was performed in triplicate and expressed as the mean with standard deviation of the measurements.

### Total polygalacturonase activity

The pectinase activity was determined following a miniaturized version of the Fructan Assay Kit protocol (Megazyme, Bray, Ireland) for reducing sugars. 10 µL of the culture supernatant and 10 µL of a 5 g L^−1^ polygalacturonic acid solution (PGA, buffered in 0.1 M sodium citrate, pH 4.5) were mixed and incubated for 40 min at 30 °C. The released reducing sugar ends were determined using a 4-hydroxybenzhydrazide solution as described in the Megazyme protocol and measured at 410 nm with a multimode microplate reader (Infinite M200, Tecan, Männedorf, Germany). Sample values were blanked against similarly prepared but non-incubated mock samples. One unit of total polygalacturonase activity was defined as the amount of enzyme that catalyzes the formation of one µmol of d-galacturonic acid per minute under the assay conditions. Assaying was performed in triplicate and results were plotted as means with standard deviations of replicate measurements.

### Endo-polygalacturonase activity

Endo-polygalacturonase activity was assessed following the protocol of Ortiz [36]. 8 µL of a 5 g L^−1^ polygalacturonic acid solution (PGA, buffered in 0.1 M sodium acetate, pH 4.5) and 8 µL of *A. niger* culture supernatant were mixed and incubated for 30 min at 30 °C in a microtiter plate prior to the addition of 40 µL of freshly prepared ruthenium red working solution (Sigma-Aldrich, 1.125 mg mL^−1^ in ddH_2_O) and 100 µL of 8 mM sodium hydroxide solution. Samples were spun down at 3200 g for 10 min. 25 µL of supernatant were mixed with 175 µL ddH_2_O in a 96-well microtiter plate and absorbance was measured at 535 nm on a microplate reader (Infinite M200, Tecan, Männedorf, Germany). Sample values were blanked against similarly prepared but non-incubated mock samples. One enzyme unit was defined as the amount of enzyme required to hydrolyze 1 µg of polygalacturonic acid into smaller fragments unable to precipitate with the dye per minute under the assay conditions. Assaying was performed in triplicate and results were plotted as means with standard deviations of replicate measurements.

### Hydrolysis of sugar beet pulp

The hydrolysis of pectin-rich residues was conducted with pre-dried (50 °C) and milled sugar beet press pulp provided by Südzucker AG (Obrigheim, Germany). 10 g of pre-sterilized pulp was mixed with 100 mL of reaction solution. The reaction solution consisted of 90 mL of sterile filtered (0.22 µm) enzyme supernatant of the strains *A. niger* N402 (172 ± 4 mg L^−1^ Protein; 20 ± 26 U L^−1^ total PGase activity) or *A. niger* ATCC 11414 (111 ± 9 mg L^−1^ Protein; 1476 ± 32 U L^−1^ total PGase activity) buffered with 10 mL of 1.0 M sodium acetate (pH 4.5). Hydrolysis was carried out at 180 rpm and 30 °C for 138 h in sterile and closed 250 mL glass bottles (DWK Life Sciences GmbH, Mainz, Germany) to prevent evaporation and contamination. Time-series samples were taken from homogenized hydrolysis mixtures and stored at − 80 °C until further use. Respective amounts of released sugars were plotted against the sampling time points and the second-order polynomial fitting curves were generated.

### HPAEC-PAD analysis of the hydrolysis supernatant

Free d-GalA and neutral sugar amounts in hydrolysis samples (diluted 1:4000) were determined on a Dionex ICS 3000 HPAEC-PAD instrument setup with a Dionex AS Autosampler, a Dionex gradient mixer GM-3 (Dionex Corp., Sunnyvale, California, USA) and a CarboPac PA1 standard bore guard column (4 × 50 mm) plus a CarboPac PA1 preparative IC column (4 × 250 mm, both Thermo Fisher Scientific Inc., Waltham, Massachusetts, USA) using a 12.5 min linear gradient of 100–250 mM sodium acetate in 100 mM sodium hydroxide solution (prepared in low total organic carbion deionized water) at 1 mL min^−1^ flow rate and constant 30 °C elution temperature.

## Results and discussion

### Shake flask-based pre-selection of *A. niger* strains for pectinase production

**Fig. 2:**
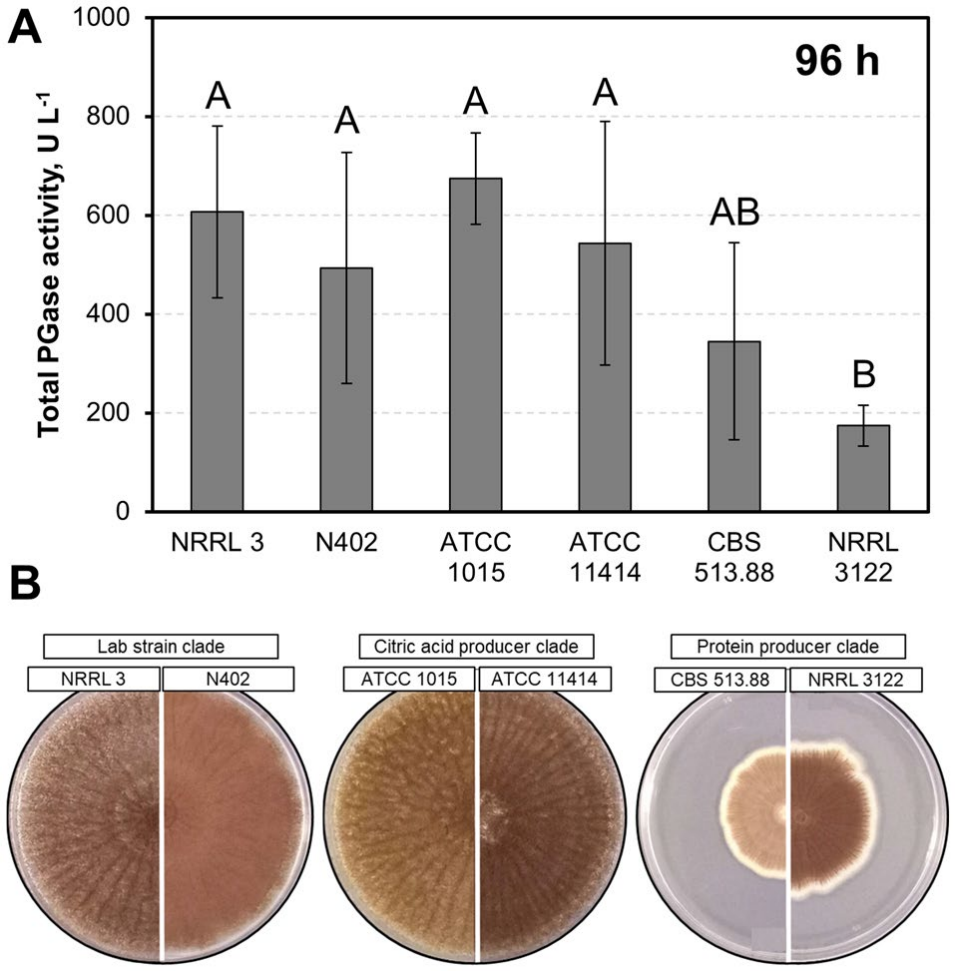
Polygalacturonase activity and sporulation density of *A. niger* strains. **a** Total PGase activity of *A. niger* culture supernatants of a representative shake flask batch after 96 h of incubation in minimal medium + 2% (w/v) pectin C, plotted as means with sample standard deviations. Different capital letters indicate significant differences within the displayed data groups (*p* < 0.05) using a one-way ANOVA followed by a Tukey’s post hoc test. Group AB showed no significant differences either to group A and group B. **b** Sporulation densities of all strains on rich complete medium after 6 days

As a first step in the identification of an ideal base strain for pectinase production among the selected *A. niger* strains, an initial fast and cost-efficient experiment was conducted in small scale using shake flasks, as typically applied in strain screenings (e.g., [40]). Total polygalacturonase (PGase) activity was measured in supernatants of *A. niger* cultures after 96 h of cultivation in minimal medium supplemented with 2% (w/v) pectin C as carbon source (Fig. 2a). Strains *A. niger* NRRL 3122 and *A. niger* CBS 513.88 repeatedly had the lowest total PGase activities of all strains tested (174 ± 41 U L^−1^ and 345 ± 199 U L^−1^, respectively), while *A. niger* NRRL 3 (607 ± 174 U L^−1^), *A. niger* N402 (493 ± 233 U L^−1^), *A. niger* ATCC 1015 (674 ± 92 U L^−1^), and *A. niger* ATCC 11414 (543 ± 246 U L^−1^) had superior total PGase activities (*p* < 0.05). Additionally, *A. niger* NRRL 3122 and *A. niger* CBS 513.88 gave lower spore densities on rich medium, which was rated as a disadvantage for larger scale liquid culture inoculations (Fig. 2b). Based on the combination of these results, strains *A. niger* NRRL 3122 and *A. niger* CBS 513.88 were excluded from further tests.

*A. niger* CBS 513.88 (and its ancestor *A. niger* NRRL 3122) differ from *A. niger* ATCC 1015 and its closely related *A. niger* NRRL 3 lineages by additional glucoamylase (*glaA*) genes acquired through horizontal gene transfer, as well as the upregulation of amino acid synthases (for those amino acids overrepresented in the GlaA protein) and their respective tRNAs [4]. Considering their poor performance in this study, however, *A. niger* CBS 513.88 and *A. niger* NRRL 3122 do not seem to have a universal advantage over the other tested strains in terms of protein production and secretion per se, but rather a limited one for GlaA expression.

### Comparison of pre-selected *A. niger* strains in submerged stirred-tank bioreactor batch cultivations

**Fig. 3:**
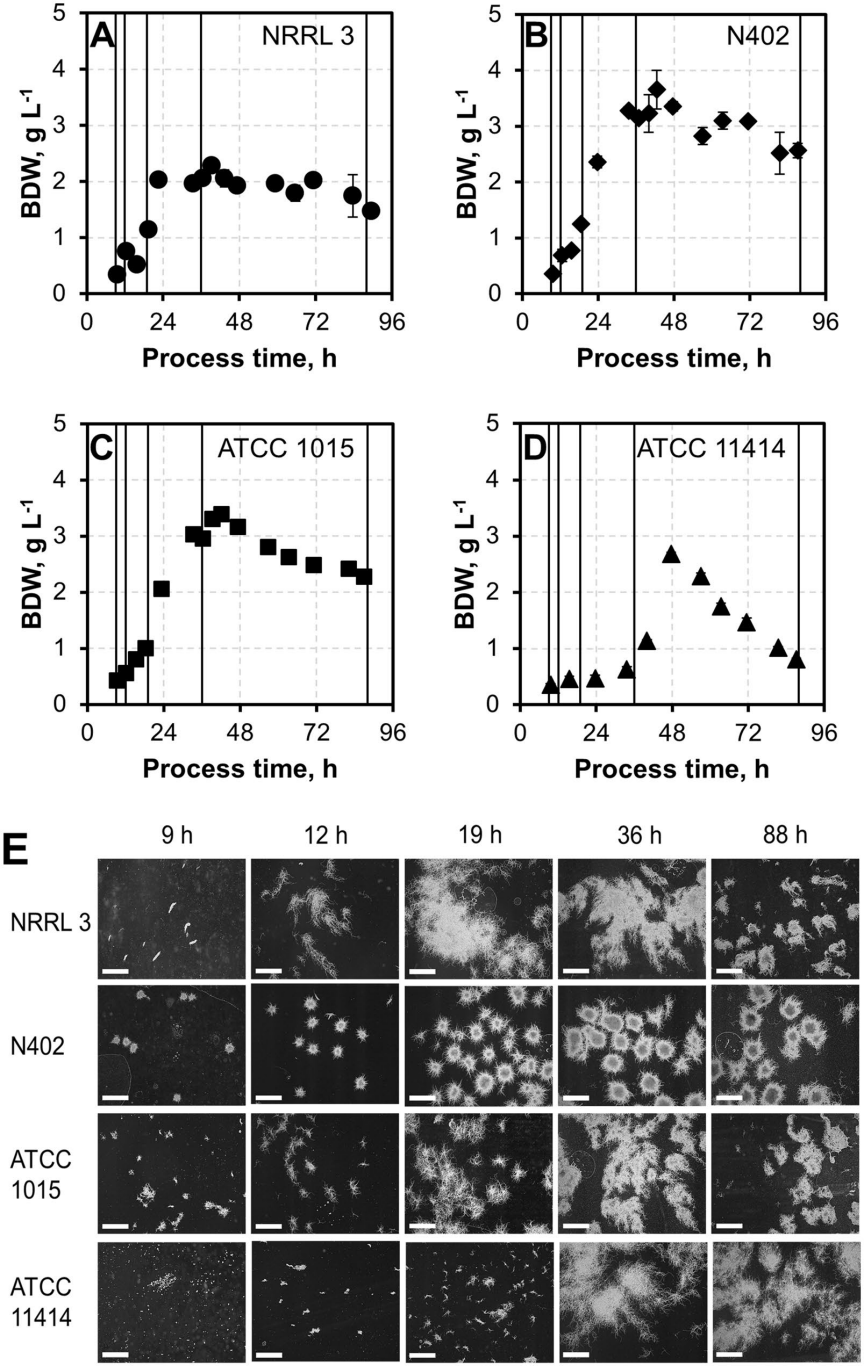
Biomass dry weight concentrations (BDW) and morphology of selected *A. niger* strains. **a**–**d** BDW of *A. niger* NRRL 3 (**a**, filled circle), *A. niger* N402 (**b**, filled diamond), *A. niger* ATCC 1015 (**c**, filled square), and *A. niger* ATCC 11414 (**d**, filled triangle) during 90 h submerged batch cultivations in a 3 L stirred-tank bioreactor with 2% pectin minimal medium, plotted as means with sample standard deviations. **e** Morphological changes of all four *A. niger* strains throughout the cultivations are shown below. White scale bars indicate 1 mm. Morphology sampling times are indicated by lines in (**a**–**d)**

**Fig. 4:**
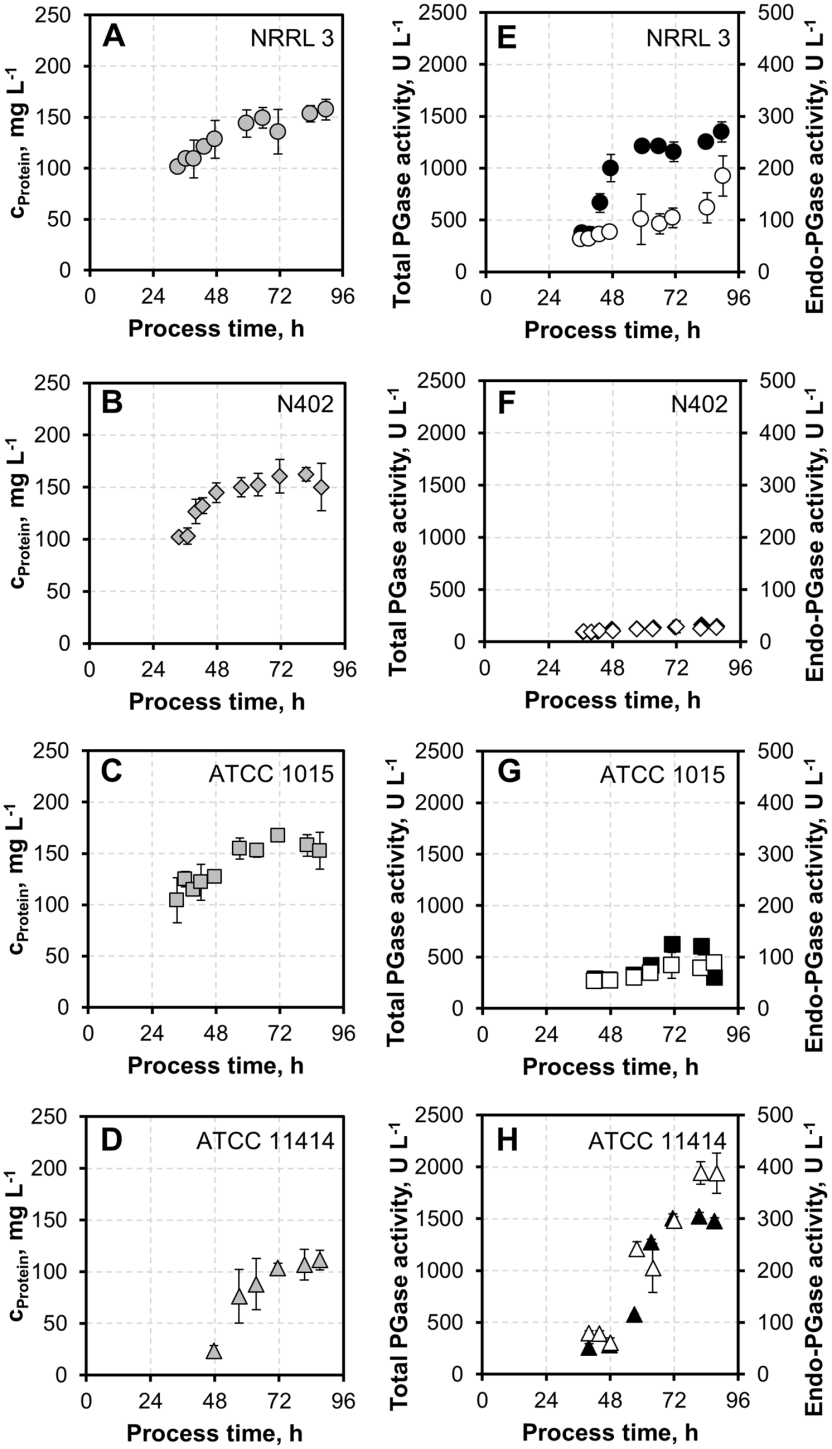
Protein concentrations, total PGase, and endo-PGase activities of pre-selected *A. niger* strains in 3 L controlled stirred-tank batches. **a**–**d** Total secreted protein concentrations (grey) in the culture supernatants of pre-selected *A. niger* strains in 2% pectin minimal medium over a time course of 90 h, plotted as means with sample standard deviations. **e**–**h** Total (black) and endo-PGase activities (white) of culture supernatants throughout the cultivation, plotted as means with sample standard deviations

A high degree of control over mechanical and physicochemical parameters influencing submerged culture morphology as well as enzyme activity is important for achieving high pectinase activity [1, 14, 54]. In the next step, pectinase production of all strains was, therefore, evaluated in batch processes on a 3 L scale in controlled stirred-tank bioreactor cultivations to perform selection under robust and reproducible conditions (see Figs. 3, 4). To this end, submerged batch cultivations in 2% (w/v) pectin minimal medium were conducted to investigate the pectinolytic properties of the four best performing *A. niger* strains from the pre-selection. For each batch cultivation, biomass dry weight concentration (BDW), total protein concentration (c_Protein_), total polygalacturonase, and endo-polygalacturonase (PGase) activity, as well as fungal morphology, were determined, all being highly relevant variables for strain productivity in submerged cultures [29, 54]. Since the level of morphology control is generally higher in stirred-tank bioreactors compared to shake flasks, we see our bioreactor approach as advantageous for in-depth strain comparison after pre-selection [14].

#### Biomass dry weight concentrations and morphology

In case of *A. niger* NRRL 3, BDW rose to a maximum of 2.28 ± 0.12 g L^−1^ during the first 39 h and slightly decreased about 35% afterwards until the end of the cultivation (1.47 ± 0.01 g L^−1^) (Fig. 3a). The BDW of the strains *A. niger* N402 and *A. niger* ATCC 1015 showed a similar behavior, increasing to 3.65 ± 0.35 g L^−1^ (*A. niger* N402) and 3.38 ± 0.07 g L^−1^ (*A. niger* ATCC 1015) within 42 h before decreasing by 30% until the end of the cultivation (Fig. 3b, c). The behavior of strain *A. niger* ATCC 11414 was different. Its BDW peaked only after 48 h (at 2.68 ± 0.03 g L^−1^), with a drastic decrease of 70% to 0.81 ± 0.02 g L^−1^ at the end of the observation period (Fig. 3d). The loss of BDW in the cultivations correlates with the dissolved oxygen concentration (DO) and the carbon dioxide fraction in the exit gas measured online. When the BDW loss started, the DO concentration increased and the carbon dioxide fraction in the exit gas decreased, even though there was no change in the aeration of the process, indicating depletion of pectin C as the sole energy source provided (data not shown).

Since the productivity of filamentous fungi in submerged cultivations depends on their morphology [13, 24, 37, 57], this parameter was monitored throughout each submerged cultivation. Figure 3e depicts the morphology of all four strains after 9, 12, 19, 36, and 88 h of the cultivation. After 36 h, all strains had a stable morphology. *A. niger* NRRL 3 and *A. niger* ATCC 11414 showed disperse, filamentous growth, *A. niger* N402 strongly pellet-like growth, and *A. niger* ATCC 1015 a less dense but still pelleted form of growth. Towards the end of the cultivation, the morphology of *A. niger* NRRL 3 shifted to a more pelleted structure. Notably, strong yellow pigmentation occurred after 34 h in all culture supernatants with the exception of *A. niger* ATCC 11414, where only mild pigmentation was observed after 58 h. The characteristic color of the pigment matches the description of Aurasperones, which are well-known side products in many *A. niger* fermentations [48]. As all four batch cultivations were run under identical conditions, these results indicate significant intra-species differences in physiological regulation among the selected strains that cannot be explained by comparison of their genomic sequences alone [4].

#### Secreted protein concentrations and PGase activities

Next, the total secreted protein concentration in the supernatants (Fig. 4a–d) as well as the total and endo-specific PGase activities were assessed (Fig. 4e–h). The protein concentration of the cultivations with *A. niger* NRRL 3, *A. niger* N402, and *A. niger* ATCC 1015 increased throughout the cultivation, peaking at 157 ± 10 mg L^−1^ (90 h), 162 ± 6 mg L^−1^ (81 h), and at 167 ± 5 mg L^−1^ (71 h), respectively. Following the observed BDW decrease towards the end of cultivation, *A. niger* N402 and *A. niger* ATCC 1015 also displayed a mild decrease in protein concentration. In accordance with its delayed biomass generation (Fig. 3d), *A. niger* ATCC 11414 showed its maximal protein titer (111 ± 10 mg L^−1^) only after 87 h (Fig. 4d). This protein concentration was 32% lower than the maximal concentration observed for the other three investigated strains. However, considering specific secretion rates (normalized to fungal biomass), *A. niger* ATCC 11414 performed *on par* with the other strains—particularly towards the end of the incubation time. Disperse mycelial growth and pellet-like growth did not correlate with the overall amount of secreted protein between different strains of *A. niger.* However, dispersed mycelial growth, as observed for strains NRRL3 and ATCC 11414, is beneficial for the secretion of specific proteins in submerged cultures of *A. niger* and other filamentous fungi compared to pellet-like growth [29, 54].

Intriguingly, in terms of PGase activities, the dispersedly growing *A. niger* NRRL 3 and *A. niger* ATCC 11414 strains showed superior performance. Total PGase activity of *A. niger* NRRL 3 increased sharply between 36 and 59 h, with a maximum activity of 1379 ± 98 U L^−1^ (90 h). Endo-PGase activity of *A. niger* NRRL 3, however, showed a moderate and continuous increase to a maximum of 185 ± 39 U L^−1^ throughout the cultivation. The increase of endo-PGase activity appeared to be decoupled from the increase of the total PGase activity, indicating differences in expressional regulation of individual pectinase classes. The highest total PGase activity was observed for *A. niger* ATCC 11414, continuously increasing throughout the cultivation to a maximum of 1524 ± 35 U L^−1^ (82 h). Endo-PGase activity peaked at 389 ± 22 U L^−1^ at 82 h. *A. niger* ATCC 11414 thus exceeded the maximal total PGase activity of NRRL 3—as the second strongest polygalacturonase producer—by 13% and the maximal endo-PGase activity by 111%. Total secreted protein as well as biomass generation, therefore, did not correlate with detected polygalacturonase activity in this study. Moreover, the different maximum activities for endo- and total PGase as well as the different time profiles of PGase activity for *A. niger* NRRL3 and *A. niger* ATCC 11414 throughout the cultivation indicate significant differences in the regulation and expression of pectinases between these two strains. Considering the lower biomass accumulations and total protein production during the cultivation of *A. niger* ATCC 11414, this strain also generated the highest specific total and endo-PGase activities.

Assuming similar total capacities of the secretory machineries in all tested *A. niger* strains, *A. niger* ATCC 11414 is, therefore, the most promising candidate for genetic improvements towards pectinase overexpression. Less off-target secondary metabolism activity (as judged by pigment formation; see Fig. S1) and highest specific polygalacturonase production hold promise for additional metabolic capacities which might be exploitable for enhanced pectinase expression.

### Comparison of hydrolytic performance of culture supernatants for d-GalA release from complex pectinaceous substrates

*A. niger* ATCC 11414 was determined as the most promising base strain for pectinase production out of six strains under the tested cultivation conditions. *A. niger* ATCC 11414 not only had the highest total and specific PGase activity, but also a disperse morphology desirable for protein secretion.

**Fig. 5:**
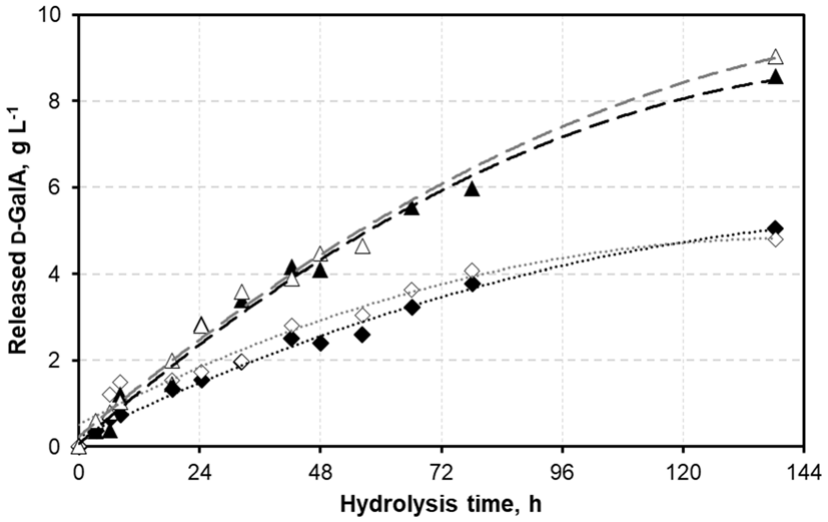
d-GalA release from 9% (w/v) sugar beet press pulp (SBPP) using *A. niger* ATCC 11414 vs. *A. niger* N402 culture supernatant. Black and white triangles (open triangle, filled triangle) represent replicates of hydrolysis using 96 h culture supernatants of *A. niger* ATCC 11414 stirred-tank batch cultivations in 2% pectin minimal medium, with respective dashed lines in grey (*y* = − 0.1545*x*^2^ + 2.411*x* + 0.2364; *R*^2^ = 0.993) and black (*y* = − 0.1772*x*^2^ + 2.4832*x* + 0.069; *R*^2^ = 0.988). Black and white diamonds (filled diamond, open diamond) represent two replicates using supernatants of N402 with respective dotted lines in black (*y* = − 0.0901*x*^2^ + 1.3532*x* + 0.2218; *R*^2^ = 0.990) and grey (*y* = − 0.122*x*^2^ + 1.4493*x* + 0.5205; *R*^2^ = 0.960)

To test whether the selection of *A. niger* ATCC 11414 based on defined substrate assay conditions would translate into improved activity also on complex pectinaceous biomass, d-GalA release from milled dry sugar beet press pulp (SBPP) using *A. niger* ATCC 11414 culture supernatant was compared against that of the *A. niger* N402 culture supernatant as the ancestor of today’s standard laboratory strains. Using a 96 h *A. niger* ATCC 11414 buffered culture, an average of 8.8 g L^−1^ of free d-GalA was released from 9% (w/v) SBPP within 138 h, as compared to an average of 4.9 g L^−1^ for *A. niger* N402 culture supernatant (Fig. 5). Taking into account the water molecules incorporated during hydrolytic cleavage of d-GalA from polymers, this corresponded to degraded amounts of 8.0 g L^−1^ and 4.5 g L^−1^ of the provided biomass, respectively. Considering a total d-GalA content in SBPP of approximately 22% (w/w) [27], ~ 36.4% of the expected d-GalA was released using *A. niger* ATCC 11414 culture supernatant (vs. ~ 20% with *A. niger* N402 culture supernatant). In other words, the same release level was obtained in less than 45% of the process time. In summary, thorough screening and activity-driven selection of *A. niger* strains from a set of readily available and highly referenced strains resulted in a 75% higher d-GalA release compared to that obtained with the standard lab strain. Additionally, higher quantities of the main sugars of SBPP (hemi-)cellulosic fractions, such as l-arabinose, d-glucose, d-fructose, and d-galactose [27], were released using *A. niger* ATCC 11414 supernatant compared to *A. niger* N402 supernatant (see Table 1, Fig. S4–7).

**Table 1:** Amount of released sugars after 138 h of hydrolysis

Strain culture supernatant used in hydrolysis	d-GalA	l-Ara	d-Glc	d-Fru	d-Gal	l-Rha	d-Xyl	d-Man
*A. niger* N402	4.9	6.8	1.8	0.9	0.7	< 0.2	< 0.2	n.d
*A. niger* ATCC 11414	8.8	7.1	2.9**	1.1	1.0*	< 0.2	< 0.2	n.d

Released sugar amounts are expressed as the average of duplicates in g L^−1^ with deviation from mean < 10%

*n.d*. non-detected sugars, *l-Ara l*-arabinose, *d-Glc d*-glucose, *d-Fru d*-fructose, *d-Gal d*-galactose, *l-Rha l*-rhamnose, *d-Xyl d*-xylose, *d-Man d*-mannose

*Deviation from mean = 11.4%, **single replicate value

Monomeric d-GalA release from SBPP of up to 79% within 48 h has been reported in highly optimized saccharification conditions using combinations of commercial pectinase mixes of the *Aspergillus* genus at enzyme concentrations comparable to those used in this study [30], while no comparable efficiency data exist for direct application of crude *Aspergillus* culture supernatants on SBPP. Furthermore, the hydrolysis setup presented in this study was used as a pectinase production benchmark in strain selection only and has not yet undergone optimization for ideal d-GalA release conditions. Additional optimization of culture conditions for *A. niger* ATCC 11414 may further improve its performance, e.g., in terms of protein secretion, for which titers of up to 20 g L^−1^ have been reported [33]. Pectins, furthermore, comprise a variety of sugars and sugar acids with a highly diverse set of linkages between them [20]. Hence, a highly complex enzyme cocktail is required to facilitate efficient sequential and synergistic removal of decorating side chains from the polygalacturonic acid backbone for subsequent d-GalA release. Media supplementation with (hemi-)cellulosic substrates and thereby induced expression of (hemi-)cellulases during pectinase production could further contribute to d-GalA release from complex biomasses [11, 30]. Research in this field is still actively ongoing, but mostly focusing on process engineering, as recently demonstrated for continuous generation of d-GalA from SBPP pectin extracts in membrane enzyme reactors [15]. Genetic engineering may provide additional means to induce (hemi-)cellulase expression for improved d-GalA release from complex pectinaceous biomasses. Via systematic screening and strain performance evaluation under controlled conditions, we complemented this research with an important comparison of highly cited, openly available and readily applied *A. niger* strains. Endeavors to use SBPP and other complex pectinaceous biomasses of interest for industrial d-GalA supply in the context of the bio-economy, hence, could highly benefit from this work.

## Conclusion

Considering the lack of systematic screening for *A. niger* strains with high pectinase production, we have implemented a robust protocol for the discrimination of competing strains in controlled stirred-tank bioreactors. Superior performance of *A. niger* ATCC 11414 was verified in a realistic setting using complex sugar beet press pulp. This strain shows potentially untapped metabolic and secretory reservoirs that could be exploited for improved pectinase production via targeted genetic engineering. However, to foster transfer of research results to industrial applications, it will be necessary to establish genetic tools, such as non-homologous end-joining suppressors or genetic markers, in this non-standard host strain.

E-supplementary data of this work can be found in the online version of the paper.

## Electronic supplementary material

Below is the link to the electronic supplementary material.

Supplementary file1 Fig. S1: Yellow pigment formation in *A. niger* culture supernatants. Accumulation of yellow pigmentation in culture supernatants of *A. niger* NRRL3, N402, ATCC1015 and ATCC11414 submerged stirred tank bioreactor batch cultivations in 2% pectin minimal medium. (TIF 1877 kb)

Supplementary file2 Fig. S2: Reproduced submerged stirred tank batch cultivations of *A. niger* N402 and *A. niger* ATCC 11414. Biomass dry weight concentrations (**a**, **b**), total protein concentration (**c**) and total PGase activity (**d**) of the reproduced cultivations of *A. niger* N402 (filled diamond) and *A. niger* ATCC 11414 (filled triangle). Grey symbols display the values of the cultivations mentioned in the results and discussion. (TIF 308 kb)

Supplementary file3 Fig. S3: Specific protein concentrations of selected *A. niger* strains. *A. niger* NRRL 3 (**a**, filled circle), *A. niger* N402 (**b**, filled diamond), *A. niger* ATCC 1015 (**c**, filled square) and *A. niger* ATCC 11414 (**d**, filled triangle) specific protein concentrations normalized by BDW during 90 h submerged batch cultivations in a 3 L stirred tank bioreactor in 2% pectin minimal medium. (TIF 295 kb)

Supplementary file4 Fig. S4: L-arabinose release from 9% sugar beet press pulp (SBPP) using *A. niger* ATCC 11414 vs. *A. niger* N402 culture supernatant. Black and white triangles (open triangle, filled triangle) represent replicates of hydrolysis using 96 h culture supernatants of *A. niger* ATCC 11414 stirred-tank batch cultivations in 2% pectin minimal medium, with respective dashed lines in grey (*y* = − 0.1378*x*^2^ + 2.0842*x* − 0.3886;* R*² = 0.964) and black (*y* = − 0.1745*x*^2^ + 2.3955*x* − 0.4715;* R*² = 0.977). Black and white diamonds (filled diamond, open diamond) represent two replicates using supernatants of N402 with respective dotted lines in black (*y* = − 0.0048*x*^2^ + 1.3076*x* − 0.215; *R*² = 0.997) and grey (*y* = − 0.0481*x*^2^ + 1.3925*x* + 0.0902;* R*² = 0.994). (TIF 2185 kb)

Supplementary file5 Fig. S5: D-glucose release from 9% sugar beet press pulp (SBPP) using *A. niger* ATCC 11414 vs. *A. niger* N402 culture supernatant. Black and white triangles (open triangle, filled triangle) represent replicates of hydrolysis using 96 h culture supernatants of *A. niger* ATCC 11414 stirred tank batch cultivations in 2% pectin minimal medium, with respective dashed lines in grey (*y* = 0.0453*x*^2^ + 0.5357*x* − 0.0448;* R*² = 0.954) and black (*y* = − 0.0679*x*^2^ + 0.9492*x* − 0.2538;* R*² = 0.945). Black and white diamonds (filled diamond, open diamond) represent two replicates using supernatants of N402 with respective dotted lines in black (*y* = − 0.0241*x*^2^ + 0.4811*x* − 0.0788; *R*² = 0.996) and grey (*y* = − 0.0146*x*^2^ + 0.3509*x* + 0.2036; *R*² = 0.929). (TIF 2185 kb)

Supplementary file6 Fig. S6: D-galactose release from 9% sugar beet press pulp (SBPP) using *A. niger* ATCC 11414 vs. *A. niger* N402 culture supernatant. Black and white triangles (open triangle, filled triangle) represent replicates of hydrolysis using 96 h culture supernatants of *A. niger* ATCC 11414 stirred tank batch cultivations in 2% pectin minimal medium, with respective dashed lines in grey (*y* = − 0.0435*x*^2^ + 0.4411*x* − 0.0222;* R*² = 0.948) and black (*y* = − 0.046*x*^2^ + 0.4297*x* − 0.0008;* R*² = 0.981). Black and white diamonds (filled diamond, open diamond) represent two replicates using supernatants of N402 with respective dotted lines in black (*y* = − 0.018*x*^2^ + 0.2303*x* - 0.0102;* R*² = 0.998) and grey (*y* = − 0.0322*x*^2^ + 0.3022*x* + 0.035;* R*² = 0.990). (TIF 2190 kb)

Supplementary file7 Fig. S7: D-fructose release from 9% sugar beet press pulp (SBPP) using *A. niger* ATCC 11414 vs. *A. niger* N402 culture supernatant. Black and white triangles (open triangle, filled triangle) represent replicates of hydrolysis using 96 h culture supernatants of *A. niger *ATCC 11414 stirred tank batch cultivations in 2% pectin minimal medium, with respective dashed lines in grey (*y* = − 0.0423*x*^2^ + 0.4679*x* − 0.1405;* R*² = 0.954) and black (*y* = − 0.0233*x*^2^ + 0.3521*x* − 0.1109;* R*² = 0.892). Black and white diamonds (filled diamond, open diamond) represent two replicates using supernatants of N402 with respective dotted lines in black (*y* = − 0.0204*x*^2^ + 0.2856*x* − 0.1176;* R*² = 0.954) and grey (*y* = − 0.0342*x*^2^ + 0.3475*x* + 0.0389;* R*² = 0.973). (TIF 2188 kb)
