# Supplementary figures and images for: Comparative evaluation of Aspergillus niger strains for endogenous pectin-depolymerization capacity and suitability for d-galacturonic acid production
Source: Bioprocess Biosyst Eng. 2020 Apr 23;43(9):1549–60. doi: 10.1007/s00449-020-02347-z (PMC7378126; doi:10.1007/s00449-020-02347-z)

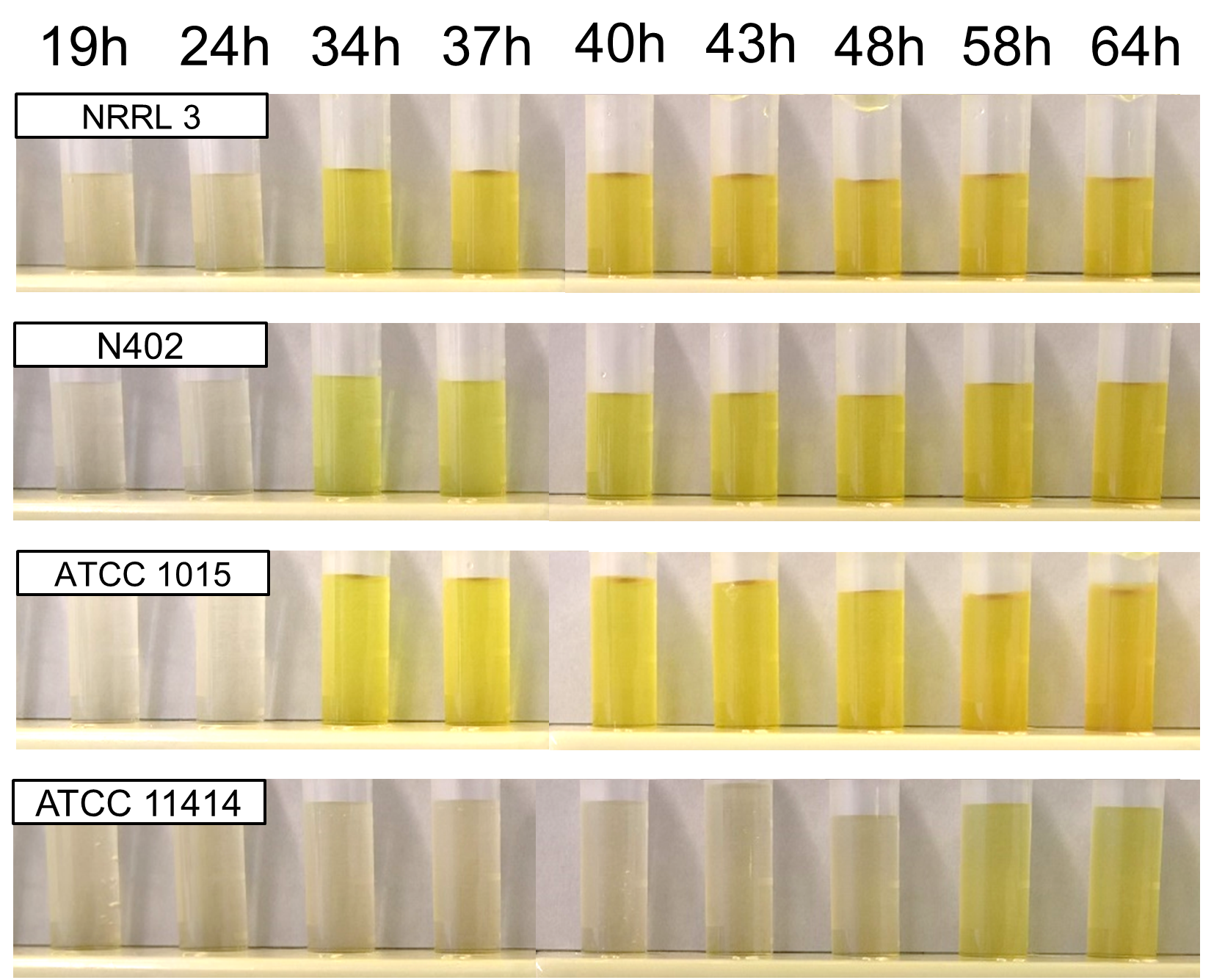

Supplement: Supplementary file 1 — Supplementary file1 Fig. S1: Yellow pigment formation in A. niger culture supernatants. Accumulation of yellow pigmentation in culture supernatants of A. niger NRRL3, N402, ATCC1015 and ATCC11414 submerged stirred tank bioreactor batch cultivations in 2% pectin minimal medium. (TIF 1877 kb) [file 449_2020_2347_MOESM1_ESM.tif]

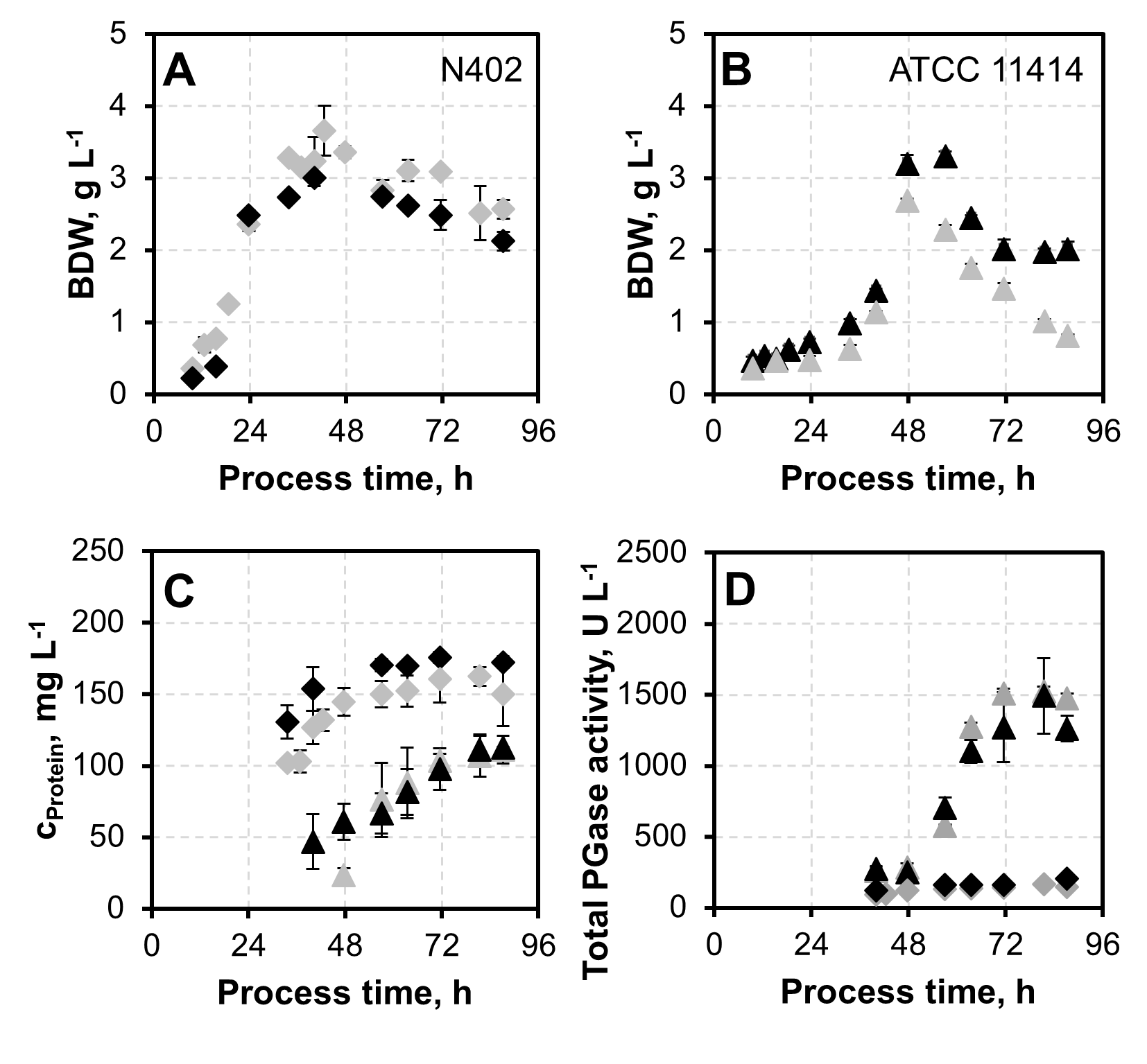

Supplement: Supplementary file 2 — Supplementary file2 Fig. S2: Reproduced submerged stirred tank batch cultivations of A. niger N402 and A. niger ATCC 11414. Biomass dry weight concentrations (a, b), total protein concentration (c) and total PGase activity (d) of the reproduced cultivations of A. niger N402 (filled diamond) and A. niger ATCC 11414 (filled triangle). Grey symbols display the values of the cultivations mentioned in the results and discussion. (TIF 308 kb) [file 449_2020_2347_MOESM2_ESM.tif]

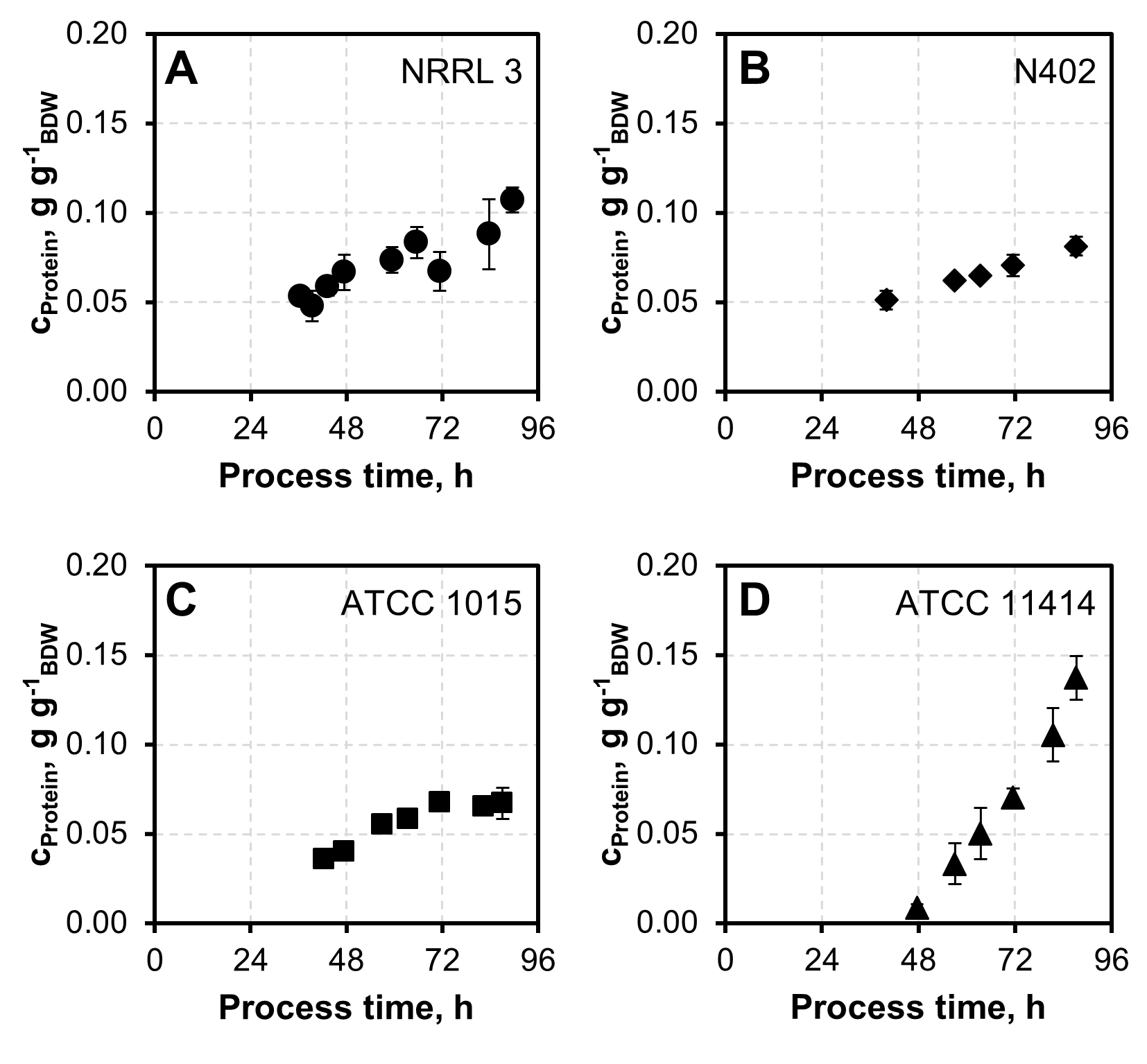

Supplement: Supplementary file 3 — Supplementary file3 Fig. S3: Specific protein concentrations of selected A. niger strains. A. niger NRRL 3 (a, filled circle), A. niger N402 (b, filled diamond), A. niger ATCC 1015 (c, filled square) and A. niger ATCC 11414 (d, filled triangle) specific protein concentrations normalized by BDW during 90 h submerged batch cultivations in a 3 L stirred tank bioreactor in 2% pectin minimal medium. (TIF 295 kb) [file 449_2020_2347_MOESM3_ESM.tif]

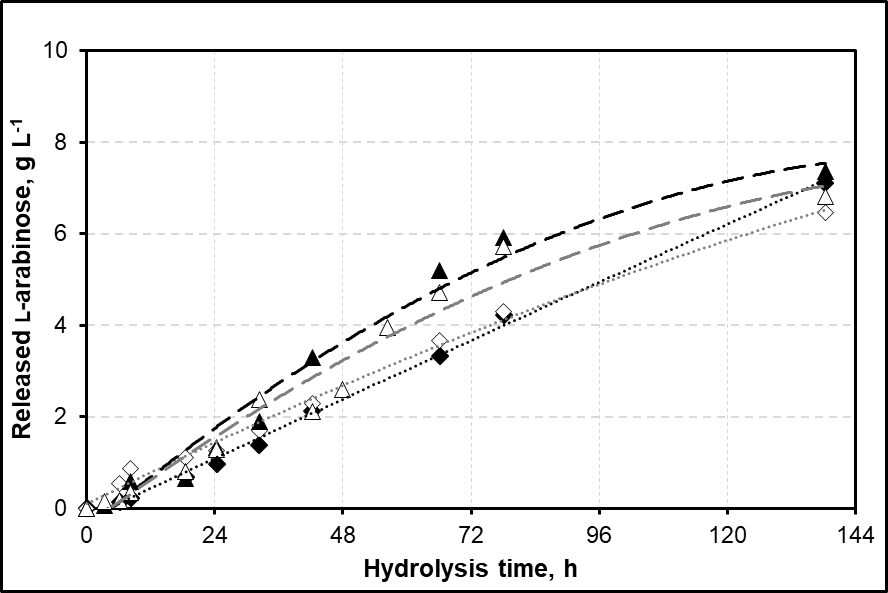

Supplement: Supplementary file 4 — Supplementary file4 Fig. S4: L-arabinose release from 9% sugar beet press pulp (SBPP) using A. niger ATCC 11414 vs. A. niger N402 culture supernatant. Black and white triangles (open triangle, filled triangle) represent replicates of hydrolysis using 96 h culture supernatants of A. niger ATCC 11414 stirred-tank batch cultivations in 2% pectin minimal medium, with respective dashed lines in grey (y = − 0.1378x2 + 2.0842x − 0.3886; R² = 0.964) and black (y = − 0.1745x2 + 2.3955x − 0.4715; R² = 0.977). Black and white diamonds (filled diamond, open diamond) represent two replicates using supernatants of N402 with respective dotted lines in black (y = − 0.0048x2 + 1.3076x − 0.215; R² = 0.997) and grey (y = − 0.0481x2 + 1.3925x + 0.0902; R² = 0.994). (TIF 2185 kb) [file 449_2020_2347_MOESM4_ESM.tif]

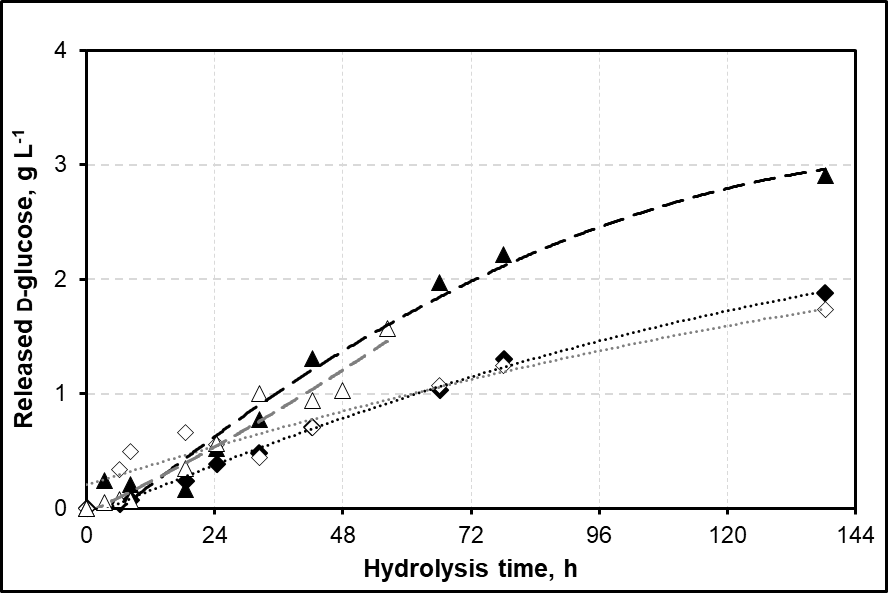

Supplement: Supplementary file 5 — Supplementary file5 Fig. S5: D-glucose release from 9% sugar beet press pulp (SBPP) using A. niger ATCC 11414 vs. A. niger N402 culture supernatant. Black and white triangles (open triangle, filled triangle) represent replicates of hydrolysis using 96 h culture supernatants of A. niger ATCC 11414 stirred tank batch cultivations in 2% pectin minimal medium, with respective dashed lines in grey (y = 0.0453x2 + 0.5357x − 0.0448; R² = 0.954) and black (y = − 0.0679x2 + 0.9492x − 0.2538; R² = 0.945). Black and white diamonds (filled diamond, open diamond) represent two replicates using supernatants of N402 with respective dotted lines in black (y = − 0.0241x2 + 0.4811x − 0.0788; R² = 0.996) and grey (y = − 0.0146x2 + 0.3509x + 0.2036; R² = 0.929). (TIF 2185 kb) [file 449_2020_2347_MOESM5_ESM.tif]

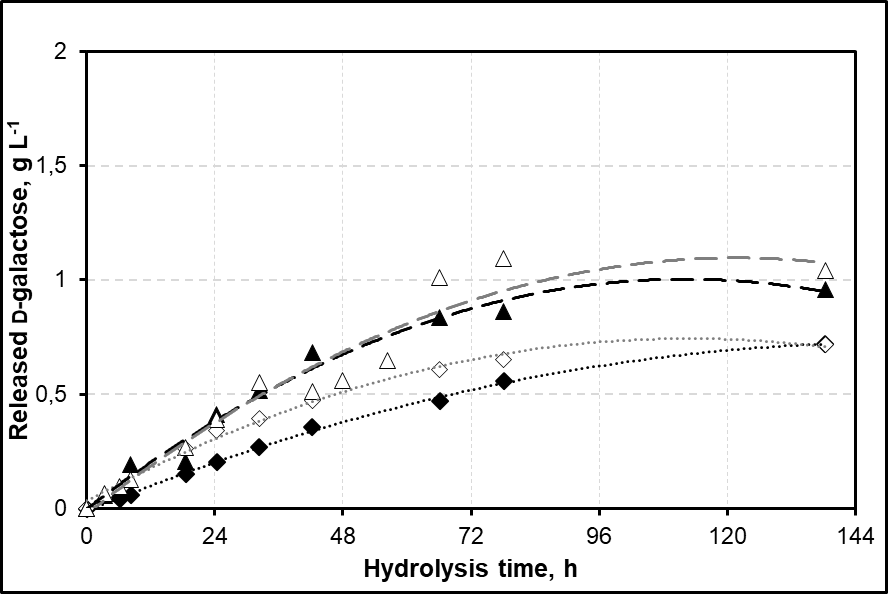

Supplement: Supplementary file 6 — Supplementary file6 Fig. S6: D-galactose release from 9% sugar beet press pulp (SBPP) using A. niger ATCC 11414 vs. A. niger N402 culture supernatant. Black and white triangles (open triangle, filled triangle) represent replicates of hydrolysis using 96 h culture supernatants of A. niger ATCC 11414 stirred tank batch cultivations in 2% pectin minimal medium, with respective dashed lines in grey (y = − 0.0435x2 + 0.4411x − 0.0222; R² = 0.948) and black (y = − 0.046x2 + 0.4297x − 0.0008; R² = 0.981). Black and white diamonds (filled diamond, open diamond) represent two replicates using supernatants of N402 with respective dotted lines in black (y = − 0.018x2 + 0.2303x - 0.0102; R² = 0.998) and grey (y = − 0.0322x2 + 0.3022x + 0.035; R² = 0.990). (TIF 2190 kb) [file 449_2020_2347_MOESM6_ESM.tif]

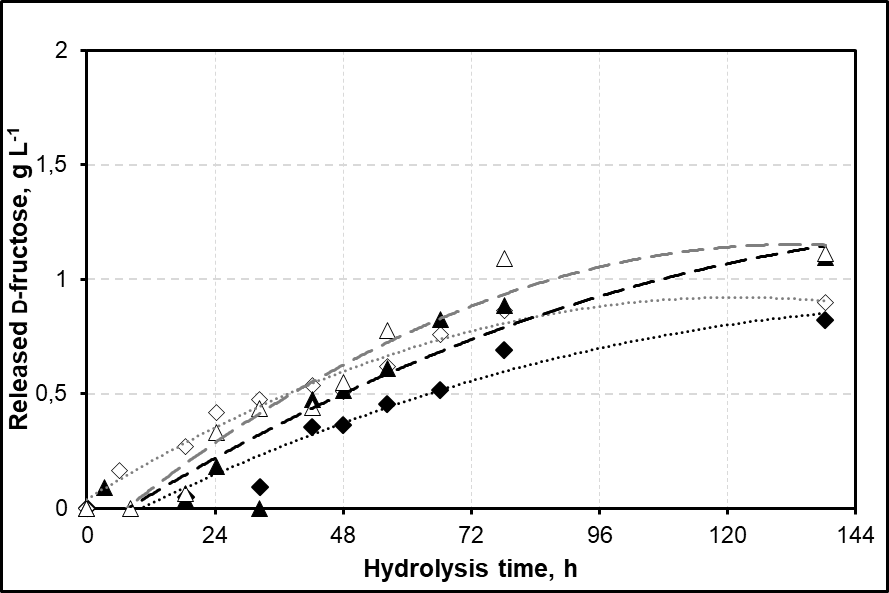

Supplement: Supplementary file 7 — Supplementary file7 Fig. S7: D-fructose release from 9% sugar beet press pulp (SBPP) using A. niger ATCC 11414 vs. A. niger N402 culture supernatant. Black and white triangles (open triangle, filled triangle) represent replicates of hydrolysis using 96 h culture supernatants of A. niger ATCC 11414 stirred tank batch cultivations in 2% pectin minimal medium, with respective dashed lines in grey (y = − 0.0423x2 + 0.4679x − 0.1405; R² = 0.954) and black (y = − 0.0233x2 + 0.3521x − 0.1109; R² = 0.892). Black and white diamonds (filled diamond, open diamond) represent two replicates using supernatants of N402 with respective dotted lines in black (y = − 0.0204x2 + 0.2856x − 0.1176; R² = 0.954) and grey (y = − 0.0342x2 + 0.3475x + 0.0389; R² = 0.973). (TIF 2188 kb) [file 449_2020_2347_MOESM7_ESM.tif]
